# Calls to a teratogen information service regarding potential exposures in pregnancy and breastfeeding

**DOI:** 10.1186/s40360-016-0076-7

**Published:** 2016-07-23

**Authors:** Sarah C. Campbell, Tyler T. Kast, Manijeh Kamyar, Julia Robertson, Catherine M. Sherwin

**Affiliations:** Nelson Laboratories, Salt Lake City, UT USA; Department of Pharmacology and Toxicology, College of Pharmacy, University of Utah, Salt Lake City, UT USA; College of Pharmacy, University of Utah, Salt Lake City, UT USA; Maternal-Fetal Medicine, Department of Obstetrics and Gynecology, School of Medicine, University of Utah, Salt Lake City, UT USA; Utah Department of Health, Pregnancy Risk Line, Salt Lake City, UT 84108 USA; Division of Clinical Pharmacology, Department of Pediatrics, School of Medicine, University of Utah, Salt Lake City, UT USA

**Keywords:** Pregnancy, Breastfeeding, Teratogen, Birth defects, Pharmaceuticals, Vaccines

## Abstract

**Background:**

MotherToBaby Utah is a teratogen information service that provides support for pregnant and breastfeeding women and healthcare providers regarding risks of exposures to medications, infections, herbals, homeopathic and dietary medications, chemicals and other substances. Calls are anonymous and free of charge. This study was undertaken to examine the volume and classification of calls regarding exposures during pregnancy and breastfeeding.

**Methods:**

Data were extracted from calls requesting information about medication use and other exposures to pregnant and breastfeeding women, between January 1 2009 and December 31 2012. Descriptive statistics were calculated.

**Results:**

A total of 27,299 calls regarding 46,031 exposures were identified in this study population. The majority of calls were made by the exposed individual (82.1 %); 13.0 % were made by a healthcare provider and 4.9 % were made by a family member or acquaintance. The majority of calls concerned pregnancy (65.8 %) versus breastfeeding (34.2 %). Exposure during the current pregnancy was the subject of 88.6 % of calls. For calls where trimester information was available, the percentage of calls for first, second and third trimesters were 44.1, 32.5 and 23.4 %, respectively.

**Conclusion:**

This study found analgesics, cold medications, herbals, homeopathic, and dietary medications were of the topic of concern for the majority of the calls regarding exposure during pregnancy and/or breastfeeding. Teratogen information services gather and provide important educational resources for both patients and healthcare providers. As the majority of calls concern nonprescription drugs and vaccines, these data provide insight into a lack of education on these subjects that should be addressed during prenatal care.

## Background

Medications are commonly used during pregnancy and breastfeeding. However, there is a scarcity of data in this population published in the United States of America (USA) regarding specific exposures and their prevalence. While it has been reported that 77–96 % of pregnant women are prescribed at least one medication during their pregnancy [1–5], the majority of the available literature focuses on prescription medications. Irvine et al. [2], have reviewed the use of over-the counter antacids, oral iron, and folic acid preparations, but those drugs were not the focus of their overall study. One study has looked to review frequently consumed herbal remedies during pregnancy, both alone and concomitantly with prescribed medications to assess side effects to the mother and fetus/neonate. Generally, there is a lack of basic knowledge as to the indications for use and safety of herbal medicines used in pregnancy and breastfeeding, not only for pregnant woman, but also for clinicians.

MotherToBaby Utah is an affiliate of the national MotherToBaby service of the Organization of Teratology Information Specialists (OTIS). The goal of MotherToBaby/OTIS is to provide up-to-date and evidence-based information to pregnant and breastfeeding women, health care providers, and the general public regarding risks or relative safety of medications and other exposures during pregnancy and breastfeeding. All calls made to MotherToBaby/OTIS are anonymous, with more than 100,000 women and their health care providers seeking information about birth defect prevention every year.

There is a great need for more information regarding the use of medications in pregnancy and breastfeeding [6]. It is well known that use of medications in pregnancy and breastfeeding is widespread and medically necessary, but there is little information about which medications or exposures are the most common, especially in the United States [7]. The purpose of this study was to expand the current knowledge about the prevalence and classification of medications or exposures in pregnant and women breastfeeding in the United States.

## Methods

A retrospective review of the MotherToBaby Utah database was conducted for calls made to the MotherToBaby Utah service between January 1 2009 and December 31 2012. MotherToBaby Utah is an affiliate of MotherToBaby and is based in Salt Lake City, Utah within the Utah Department of Health [8]. Calls are taken Monday through Friday between business hours, 9–5 pm MST. Calls are also routed from nine additional states (Colorado, Idaho, Minnesota, Montana, Nevada, North Dakota, South Dakota, Virginia, and Wyoming) across the United States as well as those from within Utah. The study qualified as an expedited review as designated by the University of Utah, Utah Department of Health and IHC Internal Review Boards (IRB). Data was obtained from a de-identified database, and no consent was deemed necessary. No individual are data presented herein.

Data were collected during each call using a standardized questioning procedure throughout the call and a brief survey taken at the end of each call. Data collected during calls includes: caller identity (i.e., exposed individual, health care provider, family member, etc.), gestational age if pregnant, and age of infant if breastfeeding, whether the subject’s potential exposure was during a current, prospective, or retrospective pregnancy, and classification (e.g. pharmaceutical, environmental, etc.) of possible exposure. The counsellors ask about all potential exposures, not just the substance of concern that the caller enquires about. Important to note, the term “of highest concern” was used for the substances with the highest number of calls made to the center by regional callers. It specifically references the substances that were of highest concern to the callers and does not indicate a ranking by an organization. This designation was solely based on the highest number of calls each substance received. Lexicomp and the PDR (Physicians’ Desk Reference) were used as the standard pharmaceutical references to code the respective medicines categories.

All data collected from the calls were manually entered into the MotherToBaby Utah database at the time of the call. Data were then extracted into a separate Microsoft Access® database for further analysis. Descriptive statistics were performed in Excel® using this database; this included determination of the frequency of calls under each classification.

## Results

Within the review timeframe 27,299 telephone calls were made to MotherToBaby Utah (Table 1). The majority of calls came from the potentially exposed individual themselves (*n* = 22,404; 82.1 %); these were women who are trying to conceive, pregnant, or breastfeeding. This is in comparison with calls from health care professionals (*n* = 3557; 13.0 %), or family members or acquaintances (*n* = 1338; 4.9 %).

**Table 1:** List of medications and frequency of calls

Medication	Frequency	Category %	Total %
Analgesics	6327	100	13.75
Acetaminophen	3085	48.76	6.7
Opioid	1257	19.87	2.73
NSAID	1224	19.35	2.66
Other	761	12.03	1.66
Anti-Infectives	3498	100	7.6
Antibacterial	2008	57.4	4.36
Antifungal	704	20.13	1.53
Antiviral	601	17.18	1.31
Other	185	5.289	0.4
Cardiovascular medications	884	100	1.92
Anticoagulant	416	47.06	0.9
Antihypertensive	246	27.83	0.53
Cardiac	147	16.63	0.32
Lipid-lowering	36	4.07	0.078
Other	39	4.41	0.092
Cold medications	9865	100	21.44
Antihistamine	3671	37.21	7.98
Cough medications	2728	27.65	5.93
Decongestant	3466	35.13	7.53
Corticosteroids^a^	1191	100	2.59
Gastrointestinal medications	2423	100	5.26
Antacid	847	34.96	1.84
Antiemetic	496	20.47	1.08
Antidiarrheal	423	17.46	0.92
Laxative	91	3.76	0.2
Other	566	23.36	1.23
Psychiatric medications	4175	100	9.07
Antidepressant	2150	51.5	4.67
Sedative/hypnotic	962	23.04	2.09
Antipsychotic	343	8.22	0.75
Mood stabilizer	337	8.07	0.73
Stimulant	215	5.15	0.47
Other	168	4.02	0.36
Other medications	3183	100	6.91

^a^Exposure could be topical or ingested

Eighty percent (*n* = 21,839; 80 %) of the callers were inquiring whether a product, drug or medication was safe before they used it and exposed the fetus/nursing baby; thus making the potential for prevention more likely.

The overall majority of calls to MotherToBaby Utah concerned exposures in pregnancy (total number and percent of total; *n* = 14,806; 65.8 %) compared with breastfeeding (*n* = 9345; 34.2 %). Of the calls regarding pregnancy, 44.1 % (*n* = 6527) were in the first trimester, 32.5 % (*n* = 4814) were in the second trimester, and 23.4 % (*n* = 3465) were in the third trimester. Inquiries about exposures in pregnancy were either for a current pregnancy (*n* = 13,118; 88.6 %), planning for a future pregnancy (*n* = 1303; 8.8 %), or an exposure during a past pregnancy (*n* = 59; 0.4 %).

### Exposures

There were a total of 46,031 possible potential exposures recorded (Table 1) from the 27,299 total calls. Of these exposures, 31,546 (68.5 %) were prescription and over-the-counter (OTC) medications, 5597 (12.2 %) related to immunizations, 4462 (9.7 %) were related to herbals, homeopathic and dietary medications, 3935 (8.6 %) were non-medications (includes health care items; e.g., toothpaste, bubble bath, and environmental chemicals), and 491 (1.1 %) were substances of abuse. There were a total of 924 unique possible exposures. For calls classified as environmental exposures, this included alcohol, however, very few calls related to alcohol exposure. In addition, the non-medications category included beauty products, bug repellents, cigarettes/nicotine/tobacco/marijuana, hot tubs/hot baths, solvents/paint.

With regards to herbals, homeopathic, and dietary medications, using the MotherToBaby database, dietary medications are entered only by their category (i.e., herbs/dietary medications). This is a limitation of the study and the MotherToBaby operation is in process of correcting this. The risk statement for these exposures provided to the caller is the same for most inquires on herbs, homeopathic, and dietary medications: *these products are not regulated by the FDA, therefore, exact product ingredients are not known and difficult to know whether or not the product is of concern. There is not enough published data to determine whether these products are safe to use on a regular basis in pregnancy or while breastfeeding.*

The exposures for which advice was most frequently sought were for the influenza vaccination (*n* = 3422; 7.4 %), acetaminophen (*n* = 3085; 6.7 %), and pseudoephedrine (*n* = 1836; 4.0 %).

### Immunizations

Calls to MotherToBaby Utah concerning immunizations/vaccinations totaled 5597. Of these calls, 68.9 % (*n* = 3854) were related directly to a vaccination and 31.1 % (*n* = 1743) were related to a vaccine-preventable disease (Table 2). Inquiries regarding influenza and/or its vaccine (*n* = 5112) made up 91.3 % of immunization-related inquiries and 11.1 % of the overall inquiries to MotherToBaby Utah. There were 1619 callers who inquired specifically about the H1N1 influenza vaccine. Vaccine-preventable disease inquiries by disease included: Anthrax *n* = 1; H1N1 *n* = 550; Hepatitis A *n* = 37; Hepatitis B *n* = 62; Hib *n* = 1; Influenza *n* = 4433; Measles, Mumps, Rubella *n* = 90; Meningococcal *n* = 11; Pneumococcal *n* = 6; Polio *n* = 2; Rabies *n* = 2; Shingles *n* = 1 Tdap *n* = 348; Typhoid *n* = 15; Varicella *n* = 30; Yellow Fever *n* = 8. No calls were reported for Japanese encephalitis, HPV or Small Pox Vaccines.

**Table 2:** List of frequency of calls related to potential exposure to non-medications

Substance	Frequency	Category %	Total %
Herbals, homeopathic and dietary medications	4462	100	9.69
Immunizations	5597	100	12.16
Vaccine	3854	68.86	8.37
Vaccine-preventable disease	1743	31.14	3.79
Non-medications (health care items; e.g., toothpaste, bubble bath, environmental chemicals, etc.)	3935	100	8.55

### Herbals, homeopathic and dietary medications

With respect to herbals, homeopathic and dietary medications, there were a total of 4462 calls, with the majority of these inquiries being unspecified (*n* = 492), this was closely followed by inquires related to caffeine consumption (*n* = 415), zinc (*n* = 246), and vitamin C (*n* = 205) supplements. With regards to inquiries being unspecific, the caller provided the name of the herbal medication, but it was not entered into the database under the individual herbal name, only the category name. This is a limitation of the study. Inquiries concerning supplements directly related to prenatal care included questions with regards to prenatal vitamins (*n* = 179), vitamin B6 (*n* = 176), and folic acid (*n* = 143).

### Environmental chemicals

Chemicals were entered under the name of the agent the individual was exposed to (e.g., toothpaste would not be entered, but fluoride, sorbitol and the other ingredients described by the caller that were contained in the toothpaste). If the individual agent was not in the database a note would be included so that the agent would be added later. There were few environmental chemicals described in this dataset, and many potential individual agents were therefore not listed and is a limitation of the study.

## Discussion

The study presented here found that analgesics, vaccinations, cold medications (decongestants, cough medications, and antihistamine), and herbals, homeopathic and dietary medications were of most frequent concerns for callers to the MotherToBaby Utah service. The majority of inquiries were from the potentially-exposed individual, who were currently pregnant rather than from those who were breastfeeding. Most of the inquiries were related to exposures during the first trimester of pregnancy.

With regards to the high proportion of calls relating to medications obtained in the non-prescription setting, including herbal/other natural health products/supplements there have been few studies that have explored the use of herbals, homeopathic and dietary medications during pregnancy and breastfeeding. Herbal products are regulated by the Food and Drug Administration (FDA) as foods. Manufacturers are not required to do the pre- and post-marketing surveillance to determine if there is an increase in the risk of birth defects, other negative pregnancy outcomes, or side-effects in breastfed babies. Due to limited information online and from health care providers, many called the MotherToBe service seeking advice about these exposures during pregnancy and breastfeeding. With limited data on dose/amount of the exposure, a true risk assessment was not possible. Until the regulations regarding herbals and other natural products change, and more evidence based studies are undertaken, there are no known safe exposures for these products during pregnancy and breastfeeding.

Holden et al. [9], interestingly found no difference in the rates of herbals and other natural product use between pregnant and non-pregnant users. Indicating that use by pregnant women in the United States is common, with over a third of the population using one or more therapies. Of more concern, only half disclosed the use of these types of substances to their providers. It appears that use is increasingly commonplace and as outlined by a recent study [10] in Australia. Pregnant women want more personal control over their bodies and are more concerned about their own personal experience when taking a herbal medicine during pregnancy rather than clinical evidence of efficacy. Many pregnant women are turning also to Chinese medicine, but similar to conventional pharmaceuticals, Chinese medicines are not free of risk, and have the potential to cause adverse pregnancy outcomes and fetal development [11]. There is limited clinical data concerning the safety of maternal exposure to Chinese medicines and basic research and mechanistic studies of the potential teratogenicity of Chinese medicines are lacking [11]. Unfortunately, the MotherToBaby Utah service does not categorize out Chinese medicines as a separate classification, however, this is to be considered in the future as the use of these products increases.

A comparative study was undertaken by Patil et al. [12], this was also a retrospective descriptive study of pregnancy and breastfeeding related inquiries to the University of North Carolina Health Care System Drug Information Center (January 2001 to December 2010). Their review of 433 calls found that inquiries were most often made during the antepartum period (34 %), followed by the postpartum (28 %) and preconception (22 %) periods. This is in contrast to the majority (88.6 %) of inquiries to MotherToBaby Utah which were made during a current pregnancy. The study undertaken by Patil et al. [12], determined the most frequent indications for inquiries to their service were related to psychiatry and infectious disease-related medication use in pregnancy.

One reason for the difference in inquires between MotherToBaby Utah could be that the North Carolina Health Care System Drug Information Centre would mostly respond to questions from health care providers and not the public. Most teratogen information services (TIS) take a larger percent of calls from the public and exposure questions would therefore differ. Future projects would include data from all TIS’ in the United States to determine regional medication usage of medications by pregnant and breastfeeding women. There is other literature describing TIS call experiences, but not from USA teratogen information services. The only other relatively recent large analysis of an equivalent service came out of Australia which showed there was a demand for such a service especially in rural areas [13].

Medication use is common and prevalence of use during pregnancy is increasing [14]. This study provides counseling focal points for health care providers in pregnant and breastfeeding populations. The vast majority of maternal medications have an undetermined risk for birth defects or other adverse fetal outcomes because they have not been adequately studied in human pregnancy [15]. Many of the common exposures do not require a prescription and are readily accessible, and make up the greatest concern of those calling MotherToBaby Utah. There is a need for increased efforts in educating the public, especially women who are currently or thinking of becoming pregnant, of risks as well as the relative safety of common exposures. Although this study helps expand current knowledge of common exposures in pregnancy and breastfeeding in the USA, larger studies are needed. Prenatal care should encompass up-to-date and appropriate education regarding these common exposures in pregnant and breastfeeding women.

### Limitations

Overall, few studies have been published regarding medication use during pregnancy and breastfeeding. As callers to the MotherToBaby Utah service may call more than one time, prevalence could not be determined in these data. Updates to the database would need to be completed in order to determine prevalence. In addition, although it is rare that a health care provider and the mother would both call regarding the same exposure concern, it could happened. Both inquires would then be reported in the database. The current system does not account for the number of times this might happen. It is reasonable to view this as educating two separate individuals, the health care provider and the mother. Educating providers on common exposures in pregnant and breastfeeding women may reduce the need for mothers and other concerned individuals to contact the service.

In the MotherToBaby Utah system, vitamins are entered separately and would be entered by the name of the vitamin e.g., vitamin B6 is entered as Pyridoxine and vitamin B9 would be entered under folic acid. When searching the database, vitamins would not be included in the results of the database under Herbs/Dietary Supplements.

With regards to pregnant women taking OTCs, it is possible that women using OTC medications are not seeking professional advice and therefore could be more likely to call MotherToBaby Utah. Unfortunately, this study was not designed to collect data to support this statement. Most of the callers get the MotherToBaby contact information from their health care provider or are told by the health care provider to call the service. It is possible that woman using OTC medications are more likely to call the service.

Another limitation of the study is that termination of pregnancies were not considered in these statistics and it is unknown whether exposures could have led to terminations either accidentally or intentionally. A 1989 study found that calls to a teratogen information service reduced the number of abortions [16]. A recent change for the MotherToBaby Utah service was to ask any caller who brings-up abortion if they are still considering abortion after the counselling. The abortion discussion is rare among the calls received and would take some time to have enough data to be statically significant.

## Conclusion

This study found analgesics, cold medications, herbals, homeopathic and dietary medications were of the most concern for the majority of the calls regarding exposure during pregnancy and/or breastfeeding. Most inquiries were regarding exposures in a current pregnancy vs. breastfeeding, specifically during the first trimester of pregnancy. This study provides focal points for public and patient education in regards to common exposures among pregnant and breastfeeding women. As the majority of calls concern non-prescription drugs and vaccines, these data provide insight into a lack of education for these exposures that should have been addressed during prenatal care. Prenatal care should encompass appropriate education and guidance on these common exposures for pregnant and/or breastfeeding women.

## Abbreviations

OTC, over-the-counter; OTIS, organization of teratology information specialists; TIS, teratogen information services
